# MRI markers of idiopathic normal pressure hydrocephalus in a population study with 791 participants: Exploring reference values and associations

**DOI:** 10.1177/19714009241303132

**Published:** 2024-12-09

**Authors:** Clara Constantinescu, Doerthe Ziegelitz, Carsten Wikkelsø, Silke Kern, Daniel Jaraj, Lina Rydén, Eric Westman, Ingmar Skoog, Mats Tullberg

**Affiliations:** 1 Hydrocephalus Research Unit, Department of Clinical Neuroscience, Institute of Neuroscience and Physiology, Sahlgrenska Academy, 195564 University of Gothenburg, Sweden; 2 Department of Radiology, Institute of Clinical Sciences, Sahlgrenska Academy, 156329 University of Gothenburg, Sweden; 3 Region Västra Götaland, Department of Neuropsychiatry, Sahlgrenska University Hospital, Sweden; 4 Neuropsychiatric Epidemiology Unit, Department of Psychiatry and Neurochemistry, Institute of Neuroscience and Physiology, Sahlgrenska Academy, 195564 Center for Ageing and Health (AGECAP) at the University of Gothenburg, Sweden; 5 Department of Neurobiology, Care Sciences and Society, Division of Clinical Geriatrics, Center for Alzheimer Research, 548198 Karolinska Institute, Sweden

**Keywords:** Idiopathic normal pressure hydrocephalus, imaging biomarker, magnetic resonance imaging, discriminatory power

## Abstract

**Purpose:**

Epidemiological studies on idiopathic normal pressure hydrocephalus (iNPH) imaging markers and their normal values are scarce. This population-based study aimed to analyze several morphologic and volumetric iNPH-related imaging markers in a large sample, determining their distribution, diagnostic accuracy, suggested cut-offs, and associations with iNPH symptoms.

**Methods:**

This cross-sectional study included 791 70 year olds, 40 with radiologically probable iNPH (iNPH_Radiol_) and 751 without iNPH features (reference). MRI measures included Evans index (EI), z-EI, brain per ventricle ratio at anterior (BVR_AC_) and posterior commissures (BVR_PC_), sulcal compression, Sylvian fissure enlargement, callosal angle, diameter of temporal horns, 3^rd^ and 4^th^ ventricles, midbrain, and pons. Volumes of ventricles, corpus callosum, and brainstem were computed using automated segmentation. ROC analysis determined imaging markers’ cut-offs. Symptoms were evaluated clinically and through self-report.

**Results:**

In the reference group, median values (95% CI) for imaging markers were as follows: EI: 0.27 (0.26–0.27), z-EI: 0.28 (0.26–0.31), BVR_AC_: 1.69 (1.48–1.90), and BVR_PC_: 2.66 (2.24–3.27). Most imaging markers differed significantly between iNPH_Radiol_ and the reference. Lateral ventricle volumes correlated better with z-EI and BVR than EI (Rs > 0.81 vs 0.68). Optimal cut-off values for z-EI, and BVR_AC_ and BVR_PC_ for distinguishing iNPH_Radiol_ were 0.32, 1.36, and 1.83, respectively. Clinical symptoms correlated moderately with imaging markers (Rs < 0.49 for iNPH_Radiol_, *p* < .01).

**Conclusions:**

We report population-based reference values and propose cut-offs for iNPH-related imaging markers and volumetric measurements. Z-EI and BVR are likely superior markers for assessing ventricular enlargement in iNPH. Imaging markers of iNPH correlate moderately with iNPH symptoms.

## Background

Although treatment of idiopathic normal pressure hydrocephalus (iNPH) with shunt surgery improves symptoms in >80% of patients,^1^ the condition remains underdiagnosed and undertreated.^2,3^ A recent population-based study showed that ∼1.5% of individuals aged 70 years have possible iNPH, suggesting that iNPH is more common than previously reported.^4^ Currently there are two guidelines for diagnosing iNPH, the Japanese Guidelines, last updated in 2021 (third edition),^5^ and the International Guidelines (I.G.)^6^ from 2005. For a diagnosis of possible iNPH both require ventricular enlargement as defined by an Evans’ Index (EI) > 0.3 and ≥1 of gait disturbance, cognitive decline, and urinary incontinence. Key differences include that the Japanese guidelines require an age of 60 years or older for diagnosis, whereas the I.G. do not state a required age, and that a diagnosis of possible iNPH in the Japanese Guidelines is acceptable even if EI <0.3, if other radiological indices meet the conditions of expanded inferior horn of the lateral ventricle, such as a callosal angle <90°, z-Evans index (z-EI) >0.42,^7^ brain per ventricular ratio at the anterior commissure (BVR_AC_) < 1.0, and/or BVR at posterior commissure (BVR_PC_) < 1.5.^8^ For the diagnosis of probable iNPH, the guidelines differ more significantly (see Table A12 for a summary). Furthermore, the Japanese Guidelines include a diagnosis of definite iNPH, which is made only when objective improvement of symptoms is shown after shunt surgery. This diagnosis does exist in the I.G. but equivalates to the term “shunt responder.”

In recent decades, a multitude of linear, angular, and volumetric measurements have been suggested as potential radiologic imaging markers for distinguishing patients with iNPH from both healthy individuals and those with conditions manifesting similar clinical symptoms.^7–23^ Several novel imaging markers have been introduced in the Japanese guidelines,^5^ such as the z-EI^7^ and BVR_AC_ and BVR_PC_.^8^ Other markers, such as the diameter of the midbrain, have proved less compelling, typically because of unclear diagnostic accuracy or low sensitivity.^21,22,24–29^ Previous studies have yielded mixed results regarding the potential of individual markers such as callosal angle and EI.^8,9,11,13–15^ The relationship between morphological imaging markers and volumetric measurements has also been doubted, for example, Ambarki et al.^10^ reported that EI had limited predictive value for ventricular volume, raising doubts about its utility as a sole marker of ventricular enlargement. Conversely, Reinard et al.^16^ reported that several radiological imaging markers, including EI, showed strong correlations with total ventricular volume. These discrepancies may be attributable to different methodologies, and many studies share the limitation of small sample sizes and incomplete exploration of correlations with clinical symptoms. Thus, accurate normal values for the most common traditional and novel radiologic imaging markers and volumetric measurements of cerebrospinal fluid (CSF) spaces in adults, as well as associations between these measures and iNPH symptoms, are unclear.

We aimed to explore the distribution and gender-specific variations of 12 iNPH-related MRI markers and corresponding segmented volumetric measurements in a large population-based study of 791 participants aged 70 years, including a subset meeting criteria for radiologically probable iNPH (iNPH_Radiol_) according to I.G.^6^ This age group was selected because iNPH is prevalent among 70 year olds,^4^ and previous studies indicate that this age is the average age of onset for the disease.^30^ We investigated the diagnostic performance of morphological imaging markers for differentiating between radiological iNPH and the reference group and explored the association between morphological and volumetric imaging markers and between these imaging markers and the iNPH symptomatology.

## Methods

### Study population

Data for this cross-sectional study were obtained from the population-based and multidisciplinary epidemiological Gothenburg H70 Birth Cohort Studies.^31^ A total of 1667 residents of Gothenburg, Sweden, born in 1944 on birth dates ending with 0, 2, 5, or 8 were invited to participate. Of the 1203 (559 men and 644 women; response rate 72%) who agreed to participate and underwent clinical examination during 2014–2016,^31^ 791 (377 men and 414 women; response rate 65.8%) also underwent structural brain MRI during the same period and were included in the study.

### Ethical considerations

The Regional Ethical Review Board in Gothenburg approved the study. Informed consent was obtained from all participants or from relatives if a participant was unable to provide it. The study was performed in accordance with the Declaration of Helsinki.

### MRI acquisition

All participants were scanned on a 3.0-T Philips Achieva system (Philips Medical Systems, Best, the Netherlands) using a protocol including T1, T2, fluid-attenuated inversion recovery (FLAIR), T2*, and diffusion-weighted imaging.^31^ Only T1-weighted and FLAIR images were used for assessment in this study. T1-weighted images were acquired with the following parameters: 1.0-mm isotropic resolution sagittal slices, TR/TE = 7.2/3.2, FOV = 255 × 256 mm^2^, acquisition matrix = 250 × 250 mm, and flip angle = 9°. T1 images had a voxel size of 1 × 1 × 1 mm. The FLAIR sequence was acquired with the following parameters: 2.0-mm isotropic resolution sagittal slices, TR/TE = 4800/280, inversion recovery delay = 1650 ms, FOV = 250 × 250 mm^2^, acquisition matrix = 250 × 237 mm, and flip angle = 90°.

### MRI analysis

All T1 3D-weighted images were processed using FreeSurfer 7.2 (https://surfer.nmr.mgh.harvard.edu/) through the HiveDB system.^32^ All MRIs had sufficient quality for FreeSurfer processing. Included volumetric measurements, measured in milliliters (mL), were left and right lateral ventricles, left and right inferior lateral ventricles, third and fourth ventricles, corpus callosum posterior, brainstem, and estimated total intracranial volume (TIV). All volumetric measures were normalized for TIV. Adjustment was performed by using residuals of a least-squares–derived linear regression between the volume of interest (y) and TIV (x).^33^

### Radiologic assessment: Morphological imaging markers

The noncommercial, open-source software 3D Slicer (version 4.11.20210226 for Windows, https://www.slicer.org/)^34^ was used for image visualization and analysis of the T1-weighted and FLAIR images. The T1-weighted volume sequences were re-angled by defining a line connecting the anterior commissure (AC) and posterior commissure (PC)^11,35,36^ and generating transaxial images parallel to the AC-PC line and coronal images perpendicular to the AC-PC line.

Twelve morphological imaging markers were included in the visual assessment protocol and analyzed on all participants. EI was measured as the ratio of the largest width of the frontal horns to the largest inner diameter of the skull in the same transaxial slice.^6^ The maximum diameter of the temporal horns was recorded bilaterally on transaxial images, and the average of the bilateral measurements was registered.^37^ Transaxial images also were used to evaluate the widest diameter of the third ventricle, measured in millimeters in the center of the ventricle in the anteroposterior direction on the slice with the widest part in the inferior-superior direction.^35^

Callosal angle was analyzed on coronal T1 images perpendicular to the AC-PC line at the level of the PC.^36^ BVR was measured in the coronal plane at the level of the AC (BVR_AC_) and PC (BVR_PC_). Each BVR was defined as the maximum distance along the *z*-axis between the roof of the lateral ventricle and the inner skull, divided by the maximum z-distance of the lateral ventricle in the same slice in the hemisphere with the largest ventricle.^8^ The z-EI was measured as the ratio between the maximum z-distance of the frontal horns of the lateral ventricles divided by the maximum cranial z-distance at the midline on the coronal plane on the AC^7^ (Figure A1).

Compression of the cortical sulci was assessed on coronal and transaxial images cranial to the lateral ventricles and dorsal to the foramen of Monro. Compression was defined as no visible cerebrospinal fluid (CSF) within the sulci and graded as follows: 0, normal or wider than normal; 1, slight compression (i.e., compression of the parafalcine sulci); 2, definitive compression (i.e., obliteration of the parafalcine and high convexity sulci).^37^ The ordinal rating scale also was combined into a dichotomous variable, with compression of sulci considered present at ≥1.

Dilation of the Sylvian fissures was evaluated on coronal and transaxial images with the thalamus and the lentiform nucleus visible. The width of the Sylvian fissure was compared to that of the surrounding sulci. Dilation was graded for each side separately as follows: 0, normal/narrow; 1, mildly/moderately enlarged; 2, highly enlarged.^38^ Bilateral ratings were combined into a dichotomous variable with dilation of the Sylvian fissures considered present if one or both sides had a rating ≥1. The dichotomous grading scales of sulcal compression and Sylvian fissure dilation were used for statistical analyses.

In a mid-sagittal slice perpendicular to a reference line along the posterior aspect of the pons, the maximum anterior–posterior (A-P) diameters of the pons and the fourth ventricle were measured in millimeters.

The maximum A-P distance of the midbrain was determined on a mid-sagittal image in millimeters perpendicular to a reference line along the cerebral aqueduct (Figure A1). The original non-reformatted FLAIR sequence was used to assess for periventricular white matter changes according to the modified Fazekas scale.^39^

To test the inter-rater reliability of the visual assessment protocol, a medical doctor (C.C.) and a senior neuroradiologist (D.Z.) individually evaluated T1 images for the same 20 participants according to the protocol. C.C. reevaluated scans for 20 participants to test intra-rater reliability with an interval of 14 days between measurements. Subsequently, C.C. performed an initial screening assessment of all T1 images according to the visual assessment protocol. D.Z. re-evaluated all T1 images with an EI >0.3 from the original assessment and reviewed FLAIR images for periventricular white matter changes to determine if the MRI data met the diagnostic criteria for iNPH_Radiol_ according to I.G.^6^

### Radiologic definitions

iNPH_Radiol_ was diagnosed according to I.G.,^6^ with the following modifications: (1) a corpus callosum angle cut-off of <90°^11^; (2) temporal horn enlargement defined as a mean temporal horn width ≥6 mm^37^; and (3) no assessment of aqueductal or fourth ventricular flow void,^5^ as no flow-sensitive sagittal T2 sequences were available for analysis, although ventricular outflow was considered patent if the fourth ventricle was not dilated and aqueduct morphology appeared normal. Based on the evaluation of MRI scans, participants were allocated to one of two groups as follows: A, EI <0.3 with no ventricular enlargement (reference group); B, iNPH_Radiol_ (Table 1.) Both observers were blinded to all clinical data. Forty participants met criteria for iNPH_Radiol_.

**Table 1.** Definition of radiological groups.

Group	Reference (A)	iNPH_Radiol_ (B)
Definition	No ventriculomegaly: EI ≤0.3	Both a and b:
		a. EI >0.3
		b. No discernible CSF flow obstruction
		And at least one of the following:
		1. Enlargement of the temporal horns (≥6 mm)
		2. Periventricular signal changes not attributable to microvascular ischemic change or demyelination
		3. Callosal angle <90°

*EI,* Evans index; *iNPH,* idiopathic normal-pressure hydrocephalus; *iNPH*_*Radiol*_, radiologically probable iNPH according to modified International Guidelines.

### Clinical assessments for iNPH diagnosis

Clinical symptoms of iNPH were gait/balance disturbance according to examination or self-report, a Mini-Mental State Examination (MMSE) score <26 or less than the mean MMSE score of all participants—1 standard deviation^40^ and urinary incontinence. Definitions of clinical symptoms have been previously described in detail.^4^

### Diagnosis of iNPH

iNPH was diagnosed in accordance with the International iNPH Guidelines—as iNPH_Radiol_ (group B) together with gait and/or balance disturbance and either cognitive impairment or urinary incontinence or both, as defined above.^4,6^ Measurement of CSF opening pressure was not practically feasible and was not performed. Even if we used the same method of application of the I.G. as in two earlier epidemiologic studies describing probable iNPH,^2,41^ we realize that we have not fully adapted to the I.G.; no CSF opening pressure values were available and we used a cut-off of <90 for callosal angle and defined temporal horn enlargement as ≥6 mm (Table 1); therefore, we termed these participants possible iNPH (iNPH_Possible_).

Twelve participants fulfilled clinical criteria for possible iNPH according to I.G., termed iNPH_Possible._ In a separate analysis, the whole sample (*n* = 791) was divided into iNPH_Possible_ (*n* = 12) and non-iNPH (*n* = 778). No participants had ventricular shunts on MRI.

One participant had radiological data but no clinical data on gait/balance, cognition, or urinary incontinence. This participant was excluded from statistical analyses that required classification into the subgroups iNPH_Possible_ or non-iNPH. Thus, 40 out of 791 were iNPH_Radiol_ and 12 out of 790 were iNPH_Possible_.

Exclusion criteria were a history of severe head trauma, meningitis, or subarachnoid hemorrhage within 1 year from study inclusion. No participants eligible for iNPH diagnosis fulfilled these criteria; thus, none were excluded.

### Additional clinical assessment

All participants also were assessed using the following test: Romberg test of balance (seconds standing) (*n* = 774),^42^ 6-min walk (walking distance in meters) (*n* = 741),^43^ and four cognitive tests: immediate (*n* = 784) and delayed recall (*n* = 783) measuring attention, short-term episodic memory, verbal working memory (number of memorized items; max 12), forward digit span (*n* = 741) and reverse digit span (*n* = 769) measuring verbal working memory, attention, and executive function (measured in number of words recalled; max 9 and 8, respectively).^44^ Questionnaires addressed current dizziness problems (yes/no; *n* = 788) and dizziness/balance problems in the last 3 months (yes/no; *n* = 788) and number of falling accidents in the last year (*n* = 782).

We also created a composite iNPH index using selected assessments of gait, balance, cognition, and incontinence (30 m at self-selected walking speed, the one-leg static balance test, MMSE, and self-reported urinary incontinence on an ordinal scale). Each score was standardized into a 0–100 scale (0, worst possible performance to 100, no impairment). The composite score was calculated as the mean of the domain scores, with gait given twice the weight of the other domains in accordance with the Hellström scale^45^: $\frac{2x\;\text{Gait}+\text{Cognition}+\text{Balance}+\text{Continence}}{\text{Number}\;\text{of}\;\text{available}\;\text{domains}}$ .

### Statistical analysis

Median values and distribution were assessed using descriptive data. The Shapiro–Wilk test was used to test for normal distribution; morphological imaging markers and volumetric measurements did not fulfill criteria for normal distribution, so we used nonparametric tests for these analyses. Differences between radiologic subgroups in imaging markers, volumetric measurements, and MMSE were assessed with the Mann–Whitney U test. Differences in proportions of nominal imaging markers were calculated using the Fisher’s exact test. Spearman’s rank correlation was used to investigate associations among morphological imaging markers, of morphological imaging markers with volumetric measurements, and of clinical symptoms with morphological imaging markers and volumetric measurements. Inter- and intra-rater reliability was measured using the intra-class correlation coefficient for continuous variables. The ability of morphological radiological imaging markers to discriminate between iNPH_Radiol_ and the reference as well as between iNPH_Possible_ and non-iNPH was evaluated by calculating the area under the curve (AUC) of the receiver operating characteristic (ROC). Youden’s index was maximized to find the optimal cut-off points from the ROC curve for morphological radiological imaging markers.^46^ All statistical tests were two-sided, and statistical significance was assumed with *p*-values <0.05. Tests were performed using SPSS 29.0 (SPSS Inc., Chicago, IL).

## Results

### Sample characteristics

Details on this population were previously published.^4^ In the overall cohort, between those who underwent MRI (*n* = 791) and those who did not (*n* = 412), the MRI group had higher education levels, higher MMSE scores, and less dementia,^47^ but the proportion of women and proportion born in Sweden did not differ.^4,47^ Of the 791 participants included in the current study, 84% were born in Sweden and 90% in a Nordic country, 52% were women, and 88% had more than mandatory education.

### Morphological radiological imaging markers in subgroups

Inter-rater agreement for imaging marker assessment was strong (intra-class correlation coefficients, 0.77 –0.99), and intra-rater reliability was >0.74 for all continuous variables. For nominal variables, there was a 100% agreement for inter- and intra-rater observations. Due to test and retest data being a constant, Cohens kappa could not be reported (statistics not shown). Values for the morphological imaging markers for the reference group, iNPH_Radiol_, and iNPH_Possible_ are presented in Tables 2 and 3. Overall, compared with women, men had significantly higher values for EI and z-EI and for widths of the ventricles, temporal horn, fourth ventricle, and pons diameter (all *p* < .05). Callosal angle and BVR_AC_ and BVR_PC_ were significantly lower in men compared to women (*p* < .001), but the sexes did not differ in midbrain width or proportions of dilated Sylvian fissures or sulcal compression (Appendix Table A1). Within the iNPH_Radiol_ cohort, men had significantly larger median third ventricle width; no other morphological markers differed significantly between the sexes (Table A10). All markers except pons diameter reliably differentiated iNPH_Radiol_ from the reference group (*p* < .001) and iNPH_Possible_ from non-iNPH (*p* < .05) (Tables 2 and 3).

**Table 2.** Comparison of imaging markers between NPH_Radiol_ and the reference group.

	Reference (*n* = 751)		iNPH_Radiol_ (*n* = 40)		
	Median (IQR)	95% CI	Median (IQR)	95% CI	AUC
EI	0.27 (0.25–0.29)	0.26–0.27	0.32*** (0.31–0.35)	0.31–0.33	0.951***
Temporal horn mean (mm)	2.10 (1.55–2.90)	2.05–2.20	6.15*** (3.45–8.20)	3.45–7.05	0.891***
Callosal angle (degrees)	120.0 (112.7–127.2)	119.0–120.9	97.40*** (85.3–110.0)	89.4–104.9	0.857***
z-EI	0.28 (0.26–0.31)	0.28–0.28	0.35*** (0.33–0.38)	0.33–0.36	0.893***
3rd ventricle width (mm)	5.30 (3.80–7.10)	5.20–5.50	9.25*** (8.00–11.50)	8.40–11.10	0.886***
BVR_AC_	1.69 (1.48–1.90)	1.66–1.72	1.15*** (1.02–1.33)	1.05–1.27	0.883***
BVR_PC_	2.66 (2.24–3.27)	2.60–2.76	1.60*** (1.36–1.97)	1.46–1.7	0.891***
Midbrain maximum diameter (mm)	12.20 (11.70–12.80)	12.20–12.30	11.55*** (11.15–12.20)	11.40–12.00	0.711***
Pons maximum diameter (mm)	23.79 (22.70–24.70)	23.60–23.90	23.30 (22.00–24.50)	22.60–24.20	0.589
4th ventricle maximum diameter (mm)	10.40 (9.30–11.50)	10.30–10.60	11.45*** (10.25–12.65)	10.70–12.00	0.666***
Compression of sulci *n*/*N*	7/751	3–14	8/40***	4–14	0.595
Enlarged Sylvian fissure, right and/or left *n*/*N*	19/751	12–29	14/40***	9–20	0.662*

*AC,* anterior commissure; *AUC,* area under the curve; *BVR,* brain per ventricle ratio; *CI,* confidence interval; *EI,* Evans index; *iNPH,* idiopathic normal pressure hydrocephalus; *iNPH*_*Radiol*_, radiologically probable iNPH according to modified International Guidelines; *IQR,* interquartile range 25th–75th percentiles; *N,* number of persons within each MRI category examined for the corresponding clinical feature of NPH; *n,* number of persons within each MRI category with the corresponding clinical feature of NPH*; PC,* posterior commissure; *z-EI,* z-Evans index.

*p < .05, **p < .01, ***p < .001.

**Table 3.** Comparison of morphological imaging markers between iNPH_Possible_ and non-iNPH groups.

	Non-iNPH (*n* = 778)		iNPH_Possible_ (*n* = 12)		AUC
	Median (IQR)	95% CI	Median (IQR)	95% CI	
EI	0.27 (0.25–0.29)	0.26–0.27	0.32*** (0.31–0.35)	0.30–0.36	0.925***
Temporal horn mean (mm)	2.10 (1.55–3.05)	2.05–2.20	5.9*** (3.38–8.23)	2.85–8.25	0.880***
Callosal angle (degrees)	119.5 (111.3–127.0)	118.5–120.7	102.8*** (87.1–110.1)	86.0–110.1	0.859***
z-EI	0.28 (0.26–0.31)	0.28–0.28	0.35*** (0.36–0.36)	0.30–0.36	0.837***
3rd ventricle width (mm)	5.4 (3.80–7.20)	5.20–5.70	8.35*** (11.60–8.00)	7.80–11.60	0.866***
BVR_AC_	1.67 (1.46–1.89)	1.65–1.70	1.21*** (1.11–1.39)	1.09–1.43	0.839***
BVR_PC_	2.62 (2.18–3.25)	2.57–2.70	1.63*** (1.42–2.04)	1.34–2.24	0.880***
Midbrain maximum diameter (mm)	12.20 (11.70–12.80)	12.20–12.30	11.60** (11.15–12.05)	11.00–12.10	0.721**
Pons maximum diameter (mm)	23.70 (22.70–24.70)	23.60–23.90	23.25 (21.90–24.50)	21.80–24.50	0.575
4th ventricle maximum diameter (mm)	10.40 (9.30–11.60)	10.30–10.60	11.55* (10.55–12.40)	10.40–12.90	0.668*
Compression of sulci *n*/*N*	13/778	7–21	2/12*	0–5	0.575
Enlarged Sylvian fissure, right and/or left *n*/*N*	28/778	19–40	5/12***	2–8	0.690*

*AC*, anterior commissure; *AUC*, area under the curve; *BVR,* brain per ventricle ratio; *CI,* confidence interval; *EI*, Evans index; *iNPH*, idiopathic normal pressure hydrocephalus; *iNPH*_*Possible*_, possible iNPH according to modified International Guidelines; *IQR,* interquartile range 25th–75th percentiles; *N,* number of persons within each MRI category examined for the corresponding clinical feature of NPH; *n,* number of persons within each MRI category with the corresponding clinical feature of NPH; *PC,* posterior commissure; *z-EI* z, Evans index.

*p < .05; **p < .01; ***p < .001.

**Figure 1.**
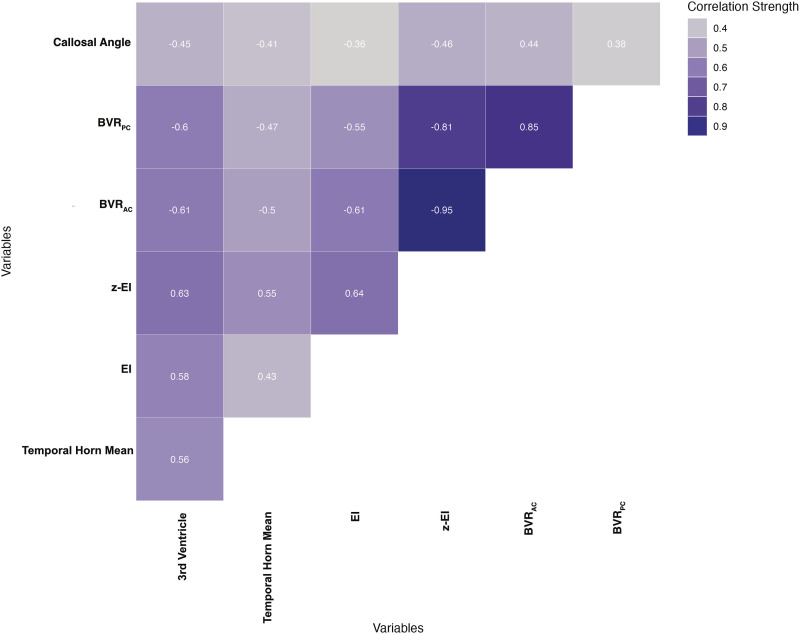
Correlation matrix of MRI-based imaging markers. Spearman correlation coefficients are shown. Shading intensity indicates strength of correlations. *BVR*_*AC*_ brain per ventricle ratio at the anterior commissure, *BVR*_*PC*_ brain per ventricle ratio at the posterior commissure, *EI* Evans index, *Temporal Horn Mean* width of the temporal horns, *3*^*rd*^ *Ventricle* mean width of the third ventricle, and *z-EI* z-Evans index.

Correlations between morphological imaging markers were strongest between z-EI, BVR_AC_, and BVR_PC_ (Figure 1).

### Volumetric measurements in subgroups

Within the whole cohort, all raw volumetric measurements except for the posterior corpus callosum were significantly greater (*p* < .001) in men than in women. All normalized volumes except for the brainstem and posterior corpus callosum differed significantly (*p* < .04) between the sexes (Table 4). In the iNPH_Radiol_ cohort, normalized volumes of the 3rd ventricle, brainstem, and total intracranial volume were significantly greater in men than in women; no other significant differences were present between the sexes (Table A11).

**Table 4.** Comparison of volumetric measurements between men and women.

	Men (*n* = 365) Median *vRaw; vNorm*	IQR *(vRaw); (vNorm)*	95% CI *(vRaw); (vNorm)*	Women (*n* = 405) Median *vRaw; vNorm*	IQR *(vRaw); (vNorm)*	95% CI *(vRaw); (vNorm)*
Left + right lateral ventricles (mL)	36.4***; 31.2**	(26.51–48.5); (23.2–40.7)	(35.3–37.7); (29.6–32.7)	26.1: 33.2	(20.2–35.2); (27.4–39.2)	(25.1–27.6); (31.7–34.1)
Left + right lateral inferior ventricles (mL)	1.4***; 1.3*	(1.1–2.0); (0.9–1.8)	(1.4–1.5); 1.2–1.3)	0.93; 1.2	(0.7–1.3);(0.9–1.5)	(0.9–1); (1.1–1.2)
3rd ventricle (mL)	2.1***; 1.9***	(1.6–2.5); 1.5–2.2)	(2.0–2.2); 1.8–1.9)	1.5; 1.7	(1.2–1.9); (1.4–2.0)	(1.4–1.5); (1.6–1.7)
4th ventricle (mL)	2.2***; 1.8*	(20.6–23.7); (1.6–2.3)	(21.9–22.5); (1.8–1.9)	1.7; 1.8	(1.4–2.1); (1.5–2.1)	(1.6–1.8); (1.7–1.9)
Brainstem (mL)	22.2***; 21.2	(20.6–23.7); (19.9–22.2)	(21.9–22.5); (21.0–21.3)	20.1; 21.0	(18.4–21.3); (19.8–22.0)	(19.8–20.3); (20.7–21.2)
Total ventricular CSF (mL)	42.1***; 36.2*	(31.6–53.8); (28.1–46.8)	(40.6–44.2); (34.5–37.7)	30.2; 37.7	(23.8–39.6); (31.7–44.8)	(29.3–32.2); (36.7–38.9)
Estimated total intracranial volume (mL)	1608.1***; NA	(1511.6–1691.5)	(1593.6–1625.2)	1411.2; NA	(1333.4–1479.5)	(1395.9–1423.1)
Corpus callosum posterior (mL)	1.0; 1.0	(0.91–1.12); (0.91–1.12)	(1.0–1.04); (1.0–1.04)	0.99; 0.99	(0.9–1.1); 0.9–1.1)	(0.96–1) (0.96–1)

*CI,* confidence interval; *CSF,* cerebrospinal fluid; *iNPH,* idiopathic normal pressure hydrocephalus; *NA,* not applicable; *IQR,* interquartile range 25th–75th percentiles; *vRaw,* raw volumes; *vNorm,* volumes normalized to intracranial volume.

* = *p* < .05; ** = *p* < .01; ***p < .001.

Among the 770 participants (35 iNPH_Radiol_ out of which 9 were iNPH_Possible_) with available volumetric measurements, all raw and TIV-normalized volumes, except for the brainstem and posterior corpus callosum, were significantly larger in iNPH_Radiol_ than in the reference group (Table 5). Between the iNPH_Possible_ and non-iNPH groups, the lateral, inferior lateral, third, and fourth ventricles and total ventricular CSF volumes were significantly larger in the iNPH_Possible_ group (Appendix Table A2).

**Table 5.** Comparison of volumetric measurements between iNPH_Radiol_ and reference groups.

	Reference (*n* = 735), median *vRaw; vNorm*	IQR *(vRaw); (vNorm)*	95% CI *(vRaw); (vNorm)*	iNPH_Radiol_ (*n* = 35) Median *vRaw; vNorm*	IQR *(vRaw); (vNorm)*	95% CI *(vRaw); (vNorm)*	∆ Volume difference, iNPH_Radiol_ vs reference, median
Left + right lateral ventricles (mL)	29.8; 31.6	(22.1–59.6); (25.2–54.8)	(28.5–30.9); (30.9–33.0)	70.5***; 60.1***	(53.3–109.6); (46.5–70.6)	(65.3–75.8); (56.5–67.6)	40.7; 28.5
Left + right lateral inferior ventricles (mL)	1.1; 1.2	(0.8–2.5); (0.9–2.4)	(1.1–1.2); (1.1–1.2)	3.1***; 2.7***	(2.2–6.1); (1.9–3.9)	(2.7–3.6); (2.2–3.2)	2; 1.5
3rd ventricle (mL)	1.7; 1.7	(1.3–2.9); (1.4–2.8)	(1.6–1.7); (1.7–1.8)	2.7***; 2.4***	(2.1–4.1); (1.9–3.1)	(2.3–3.4); (2.2–3.2)	1; 0.7
4th ventricle (mL)	1.8; 1.8	(1.5–2.9); (1.5–2.8)	(1.7–1.8); (1.7–1.8)	2.2***; 2.2**	(1.9–3.8); (1.7–2.7)	(2.2–2.7); (2.0–2.5)	0.4; 0.4
Brainstem (mL)	20.9;21.1	(19.4–25.1); (19.9–24.0)	(20.7–21.1); (21.0–21.2)	21.1; 19.1***	(18.5–25.2); (17.6–20.1)	(19.7–21.7); (18.1–19.9)	0.2; 2
Total ventricular CSF (mL)	34.5; 36.6	(26.4–67.0); (29.4–61.6)	(33.1–35.6); (35.5–37.5)	79.2***; 69.2***	(59.4–119.7); (54.7–78.7)	(74.4–85.4); (63.9–73.9)	44.7; 32.6
Estimated total intracranial volume (mL)	1483.8; NA	(1381.4–1776.8)	(1468.7–1496.7)	1669.3***; NA	(1480.5–2006.1)	(1592.0–1711.6)	185.5
Posterior corpus callosum (mL)	1.0; 1.0	(0.9–1.3); (0.9–1.3)	(0.9–1.0); (0.9–1.0)	1.1; 1.1	(0.9–1.4); (0.9–1.2)	(1.05–1.1); (1.05–1.1)	0

*CI,* confidence interval; *CSF,* cerebrospinal fluid; *iNPH,* idiopathic normal pressure hydrocephalus; *IQR, interquartile range 25th–75th percentiles*; *vRaw,* raw volumes; *vNorm,* volumes normalized to intracranial volume.

**p < .01; ***p < .001.

### Discriminatory power of morphological radiological imaging markers

EI followed by z-EI showed the highest discriminatory power between iNPH_Radiol_ and reference participants. The optimal cut-off values for selected radiological imaging markers are summarized in Table 6.

**Table 6.** Optimal cut-off values for selected morphological imaging markers.

Imaging marker	Optimal cut-off	Sensitivity (%)	Specificity (%)
EI	0.30	100	88
z-EI	0.32	83	85
BVR_AC_	1.36	80	87
BVR_PC_	1.83	75	91

*BVR*_*AC*_, brain per ventricle ratio at the anterior commissure; *BVR*_*PC*_, brain per ventricle ratio at the posterior commissure; *EI*, Evans index; *z-EI* z, Evans index.

### Associations between morphological imaging markers and volumetric measurements

The associations between z-EI and BVR_AC_ and BVR_PC_ and the normalized volumes of the lateral ventricles were very strong (Spearman’s rho (r_s_) > 0.8 for all), as were associations of morphological measurements with normalized segmented volumes of the third and fourth ventricles (r_s_ >0.8 for all). Associations between EI and normalized volumes of the lateral ventricles and between mean temporal horn width and mean volume of the inferior lateral ventricles were strong (r_s_ ≥0.7 for all). Most correlations between morphological imaging markers and the corresponding volumetric measurements were stronger with raw volumes than with TIV-normalized volumes (Table 7).

**Table 7.** Correlations of imaging markers with volumetric measurements, raw, or normalized to TIV, all *p* < .001.

Morphological imaging marker	Volumetric measurement	r_s_ *vNorm*	r_s_ *vRaw*
EI	Left + right lateral ventricle (mL)	0.539	0.680
Temporal horns mean (mm)	(Left + right inferior lateral ventricle)/2 (mL)	0.607	0.700
Callosal angle (degrees)	Total ventricular CSF (mL)	−0.197	−0.318
Callosal angle (degrees)	Posterior corpus callosum (mL)	0.177	0.177
Callosal angle (degrees)	Left + right lateral ventricle (mL)	−0.175	−0.298
z-EI	Left + right lateral ventricle (mL)	0.699	0.824
BVR_AC_	Left + right lateral ventricle (mL)	−0.680	−0.818
BVR_PC_	Left + right lateral ventricle (mL)	−0.707	−0.835
3rd ventricle width (mm)	3rd ventricle (mL)	0.809	0.911
4th ventricle width (mm)	4th ventricle (mL)	0.808	0.813
Midbrain maximum diameter (mm)	Brainstem (mL)	0.381	0.350
Pons maximum diameter (mm)	Brainstem (mL)	0.571	0.611

*BVR*_*AC*_, brain per ventricle ratio at anterior commissure; *BVR*_*PC*_, brain per ventricle ratio at posterior commissure; *EI,* Evans index; *NA,* not applicable; *TIV,* total intracranial volume; *vRaw*, raw volumes; *vNorm,* volumes normalized to intracranial volume; *z-EI* z, Evans index.

### Associations of morphological radiologic imaging markers with volumetric measurements and clinical symptoms

In the whole sample, we found significant but weak correlations between clinical symptoms and morphological imaging markers or volumetric measurements (strongest r_s_ = −0.283 for 6m gait test and brainstem volume; *p* < .05). Among iNPH_Radiol_, mean temporal horn width was moderately correlated with immediate and delayed recall measures (r_s_ = −0.486 and −0.434, respectively; *p* < .01), as were pontine width and brainstem volume with number of falling accidents in the last year (r_s_ = −0.409 and r_s_ = −0.414, respectively, *p* < .05) and total intracranial volume with urinary incontinence (r_s_ = −0.416; *p* < .05). No other clinical symptoms showed strong correlations with imaging markers or volumetric measurements (all r_s_ < 0.4), and neither did iNPH index with morphological radiologic imaging markers or volumetric measurements (all r_s_ < 0.21; see Appendix Tables A3–A9).

## Discussion

In this large, population-based study comprising a representative sample of 70 year olds, we explored the distribution of several iNPH-related morphological imaging markers and the corresponding volumetric measurements in a reference group and in people with radiological signs of iNPH or a diagnosis of possible iNPH. We report reference values and sex differences for the iNPH-specific radiologic measurements, including the novel ventricular size markers z-EI and BVR, and propose z-EI and BVR cut-off values for distinguishing radiologically probable iNPH. z-EI and BVR correlated better than EI with VV, highlighting them as likely superior imaging markers for assessing ventricular enlargement in patients with iNPH. Radiologic measurements of iNPH showed significant albeit moderate associations with iNPH clinical symptoms.

### Comparison between diagnostic guidelines

As no CSF opening pressure values were available, we termed the iNPH group possible iNPH (iNPH_Possible_) even though MRI and clinical criteria of probable iNPH according to I.G. were used for diagnosis. Had we, excluding the measurement of CSF pressure, used the Japanese Guidelines for diagnosing probable iNPH, a larger number of individuals would have been classified as fulfilling the clinical criteria of iNPH, as only gait disturbance is mandatory for diagnosis (42.5%; 17 of 40 iNPH_Radiol_ had gait and/or balance disturbance). However, fewer individuals would have fulfilled the MRI criteria of iNPH which require the neuroimaging features of tight high-convexity and medial subarachnoid spaces and enlarged Sylvian fissure associated with ventriculomegaly (DESH)^48^ as only 7.5% of iNPH_Radiol_ had DESH.

### Radiologic imaging markers

The radiologic imaging markers explored in this study were selected either because they are part of the I.G. and/or the Japanese Guidelines (EI, z-EI, BVR_AC_, BVR_PC_, sulcal compression, Sylvian fissure enlargement, callosal angle, and diameter of temporal horns) or because studies have shown that they are important from a diagnostic, predictive, or pathophysiological perspective in iNPH (3^rd 21, 35^ 4^th^ ventricles,^21^ midbrain,^22,49^ and pons^50^).

The median values of EI, z-EI, and BVR_AC_ and BVR_PC_ in this study differed in some instances from previously reported data. In a study by Jaraj et al.,^51^ the median EI for unaffected participants was similar to that for the reference group in our study (0.27 for both), but the values differ for those with iNPH_Radiol_ in our study (0.32) and their probable iNPH group (0.36). Their affected group was older with a median age of 85.5 years compared with our cohort of 70 year olds. Yamada et al reported mean values of EI: 0.34, z-EI: 0.44, BVR_AC_: 0.73, and BVR_PC_: 0.92, respectively, in an iNPH sample with a mean age of 76.9 years,^52^ and mean values of EI: 0.32, z-EI: 0.44, BVR_AC_: 0.72, and BVR_PC_: 0.89 in a different iNPH sample with a mean age of 76.7.^53^ The median EI (0.32) and z-EI (0.35) in persons with iNPH_Radiol_ in our study were lower, and BVR_AC_ (1.15) and BVR_PC_ (1.6) were notably higher compared to Yamada’s findings.

According to the Japanese Guidelines, iNPH patients typically exhibit a z-EI greater than 0.42 and BVR_AC_ and BVR_PC_ measurements of less than 1.0 and 1.5, respectively.^5^ No median values in our iNPH subgroups met these criteria, and among those with iNPH_Radiol_ 5% (2 of 40; 95% CI 0.01–1.7) had z-EI >0.42, 22.5% (9 of 40; 95% CI 0.11–0.38) had BVR_AC_ <1.0, and 37.5% (15 of 40; 95% CI 0.27–0.54) had BVR_PC_ <1.5. The studies underlying the Japanese guidelines included patients with mean ages of 76^7^ and 77 years,^8^ again older than our participants. EI increases with age and differs by sex,^51,54^ which may also be the case for these novel metrics and could explain why median values did not meet the proposed guideline cut-offs in the NPH_Radiol_ group. Cut-off values should be assessed in larger studies of patients and controls with a wider age range, preferably for different age groups.

The sexes also differed in most other radiologic markers in the current study, including findings highlighting greater raw ventricular CSF volumes in men than in women and a larger median callosal angle in women. Brix et al^54^ have proposed new, age- and sex-matched cut-off levels for EI; however, no gender- and age-matched reference values exist for novel imaging markers such as the *z*-EI and BVR_AC_ and BVR_PC_. Larger studies with a wider age range are needed that also assess sex- and age-matched reference values for these imaging markers beyond those described here for 70 year olds.

### Volumetric measurements

Participants with iNPH_Radiol_ and iNPH_Possible_ had significantly higher total VVs than the reference populations. If a proposed cut-off of >77 mL for assessing ventricular enlargement in white elderly persons^10^ is applied here, raw values for those with iNPH_Radiol_ meet this criterion, but normalized values do not, and neither raw nor normalized VVs in those with iNPH_Possible_ met the threshold. VVs vary greatly among healthy older people, and some with normal or close to normal VV may have iNPH symptoms.^10^ Gait and/or balance disturbance, cognitive impairment, and urinary incontinence are significantly associated with iNPH_Radiol_, implying symptomatic ventricular enlargement.^4^ Age,^55^ sex, and ethnicity^56–58^ also probably impact VV, and most volumetric measurements differed significantly between the sexes in the current study; the raw VV was significantly higher in men than in women (42.1 vs 30.2 mL, 95% CI: 40.6–44.2). These differences should probably be taken into account in the clinical setting.

The expansion of the bilateral ventricles in iNPH patients primarily occurs in the z-axial rather than the x-axial direction,^7,8^ and in studies by Yamada et al.^7^ and Ryska et al.,^23^ z-EI and BVR_AC_ and BVR_PC_ showed a higher discriminatory power for differentiating iNPH patients from healthy controls than EI.^7,23^ Notably, our diagnostic criteria^59^ for iNPH_Radiol_, which included EI >0.3, have probably led to a selection bias that affected our results, favoring EI. The same risk for selection bias applies to the above-mentioned studies; however, both studies use different study designs compared to our study which may explain differences in results. We consider it difficult to avoid this problem of selection bias since EI >0.3 is a signature hallmark of the present guidelines. We could have chosen to omit analyses including EI but believe that the reported results, including the specificity of EI, have relevance for the paper.

When we repeated the analysis on data for a previously described subgroup of participants with highly iNPH-specific radiological changes (*n* = 11),^4^ EI still had the highest discriminatory power (AUC = 0.98) for distinguishing iNPH_Radiol_; however, the discriminatory power of z-EI and BVR_AC_ and BVR_PC_ increased significantly to AUC 0.91, 0.91, and 0.96, respectively (data not shown), consistent with other studies.^7,23^ Caudocranial (z-EI and BVR) expansion of the lateral ventricles may indeed better discriminate iNPH from other conditions involving ventricular enlargement; thus, these markers are probably better than EI for use in the clinical setting and should be considered for inclusion in future clinical practice guidelines.

We found that the optimal cut-off values for EI, z-EI, BVR_AC_, and BVR_PC_ for distinguishing iNPH_Radiol_ were 0.30, 0.32, 1.36, and 1.83, respectively. For EI, again this cut-off and the higher sensitivity and specificity compared to z-EI and BVR are not surprising since >0.30 was used to define iNPH_Radiol_. z-EI and BVR_AC_ or BVR_PC_ showed comparable sensitivity and specificity in our sample, however as expected, lower than EI. In a future study, we plan to explore the discriminatory power of z-EI and BVR further using different cut-offs and a more holistic definition of iNPH_Radiol_ also including subjects with EI <0.3. Compared with previously suggested values,^8^ our cut-offs in this larger study are consistent with less ventricular enlargement, possibly because of our younger study sample. Cut-off values reported here could possibly be more reliable given the large population-based sample of our study.

### Associations between morphological imaging markers, and between morphological imaging markers and corresponding volumetric measurements

The novel imaging markers z-EI and BVR_AC_ and BVR_PC_ showed stronger correlations than EI with volumetric measurements of the lateral ventricles, and correlations between morphological imaging markers were the strongest for z-EI and BVR_AC_ and BVR_PC_. These results indicate that caudocranial measures (z-EI and BVR) of expansion of the lateral ventricles are superior morphologic measures of dilatation of the ventricular system compared to EI. Although raw volumes correlated more strongly than normalized volumes with morphological imaging markers, normalized values still showed a strong association. We believe that TIV-normalized values should be used when exploring volumes of CSF spaces given that ventricular volume and head size are associated.^60^ Volumetric analyses, however, are time consuming and not readily available in most hospitals, and z-EI and/or BVR_AC_ and BVR_PC_ are the most suitable indices for measuring typical iNPH radiological changes. In the aggregate, we propose that z-EI and BVR_AC_ and BVR_PC_ should be incorporated into future revisions of international diagnostic criteria for iNPH.

### Associations between imaging markers, volumetric measurements, and clinical symptoms of iNPH

Correlations between single iNPH symptoms measured as gait, balance and cognitive performance, iNPH morphological imaging markers, and volumetric measurements were weak in the whole sample and moderate in those with iNPH_Radio_. These findings indicate a weak linear relationship, possibly related to the parallel development of clinical symptoms and radiological signs of iNPH which challenges the current view of a sharp cut-off for ventriculomegaly (EI >0.3). These modest associations persisted even with adjustment of the analysis to use a composite scale (0–100, similar to the Hellström iNPH scale,^45^ rather than using each symptom alone (data not shown). As has been the consensus and reported in previous studies, the findings on the other hand indicate that clinical symptoms and ventriculomegaly do not always co-exist.^61^ Another reason for the absence of strong associations between symptoms and radiologic iNPH findings could be the use of linear correlation for assessment of associations, rather than looking at threshold effects. It is possible that iNPH symptoms do not appear or increase linearly with iNPH radiological changes, but rather that symptoms appear at first after a certain degree of radiological changes have developed. Further studies investigating symptom burden in relation to radiological cut-off values of iNPH imaging markers are needed. Having said this, we find the strongest correlations between mean temporal horn width and performance in digit span tests as well as those between measures of pontine width, brainstem volume, and fall accidents interesting. Digit span test forward and backward assesses memory function, and it is reasonable to assume that enlargement of temporal horns can influence hippocampal size and function.^62^ Fall accidents are usually associated with poor balance. Impaired postural control with retropulsion is typical in iNPH, probably caused by impaired brain stem function.^63^ These results may suggest that the CSF dynamic disturbance leading to radiological changes of iNPH cause specific clinical symptoms in different brain regions adjacent to the ventricular system.

Moreover, there is a growing consensus in the scientific community that iNPH should be recognized as a distinct form of dementia. It shares overlapping and potentially common etiopathogenetic mechanisms with other types of dementia, such as Alzheimer’s disease and frontotemporal dementia,^64^ or represents important comorbidities.^65^ Therefore, it is essential not to overlook the potential connection between NPH and other forms of dementia. Further studies are necessary to investigate reference values of radiological imaging markers in patients with diseases that overlap with NPH. Additionally, research is needed to determine cut-off values that can effectively differentiate NPH from these overlapping conditions.

## Strengths and limitations

Notable strengths of this study include the large, representative population and the use of detailed evaluation of clinical symptoms and brain MRI. In addition, an experienced neuroradiologist evaluated all MRI scans suggesting hydrocephalus. Among the limitations was the inability to consider all variables and exclusion criteria outlined in international guidelines, including confirmation of normal opening pressure; however, no participant showed radiologic signs of increased intracranial pressure. Furthermore, aqueductal flow void assessment was not possible because flow-sensitive sagittal T2 sequences were not available, although we observed no signs of aqueduct stenosis or other obstruction of CSF pathways. Consequently, no aqueductal stroke volume was measured as the necessary sequences were not available. However, aqueductal stroke volume does not have an established diagnostic value, in contrast to the other imaging markers evaluated, such as EI. The 2005 international guidelines’ radiological criteria are in some respects somewhat vague and lack a cut-off value for temporal horn enlargement. We adopted Kockum et al.'s definition of >6 mm^37^ and used a callosal angle <90° for iNPH, as recent studies suggest.^11^ We believe that these modifications do not affect the results or introduce bias but increase diagnostic accuracy.

Data on volumetric measurements were not available for all participants with iNPH_Radiol_ or iNPH_Possible,_ and their inclusion would have improved the accuracy of the ventricular volume values in these patient groups. Indeed, we had comparatively few participants with radiologic features of iNPH. Studies are needed in larger cohorts and different age groups to obtain accurate reference values for radiologic imaging markers and corresponding volumetric measurements in persons with iNPH.

## Conclusion

We examined a large, representative, population-based group of 70 year olds and report reference and sex values for several iNPH-related radiologic imaging markers and corresponding volumetric measurements, including those of a group with radiologic findings of iNPH. We suggest cut-off values for z-EI and BVR for discrimination between radiological iNPH and a reference group and conclude that z-EI and BVR are superior markers for assessing iNPH ventricular enlargement. We found moderate correlations between iNPH symptoms of gait/balance, cognition and radiologic measurements.

## Supplemental Material

Supplemental Material - MRI markers of idiopathic normal pressure hydrocephalus in a population study with 791 participants: Exploring reference values and associations Supplemental Material for MRI markers of idiopathic normal pressure hydrocephalus in a population study with 791 participants: Exploring reference values and associations by Clara Constantinescu, Doerthe Ziegelitz, Carsten Wikkelsø, Silke Kern, Daniel Jaraj, Lina Rydén, Eric Westman, Ingmar Skoog, and Mats Tullberg in The Neuroradiology Journal.
