# Supplementary material for: MRI markers of idiopathic normal pressure hydrocephalus in a population study with 791 participants: Exploring reference values and associations
Source: Neuroradiol J. 2024 Dec 9;38(4):475–87. doi: 10.1177/19714009241303132 (PMC11626555; doi:10.1177/19714009241303132)
Supplement: Supplemental Material - MRI markers of idiopathic normal pressure hydrocephalus in a population study with 791 participants: Exploring reference values and associations [file sj-pdf-1-neu-10.1177_19714009241303132.pdf]

Appendix

Title: MRI markers of idiopathic normal pressure hydrocephalus in a population study with 791 participants: exploring reference values and associations

Table A1. Comparison of imaging markers between men and women

	**Men (n=377)** mean/median	**Women (n=414)** mean/median
**Evans Index (mm)** mean/median	0.28/0.28***	0.26/0.26
**Z-EI** mean/median	0.29/0.29***	0.28/0.27
**Third ventricle (mm)** mean/median	6.74/6.70***	4.88/4.50
**Temporal horn width (mm)** mean/median	2.89/2.50***	2.22/1.90
**BVR_{AC}** mean/median	1.62/1.62***	1.77/1.74
**BVR_{PC}** mean/median	2.59/2.50***	2.85/2.77
**Callosal angle (degrees)** mean/median	114.8/116.5***	120.2/122.2
**The maximum diameter of the midbrain (mm)** mean/median	12.24/12.20	12.22/12.20
**The maximum diameter of the pons (mm)** mean/median	23.89/23.9*	23.65/23.60
**The maximum diameter of the fourth ventricle (mm)** mean/median	10.60/10.50*	10.29/10.30
**Compression of sulci** n/N	9/377	6/414
**Enlarged sylvian fissure right and/or left** n/N	19/377	14/414

BVR_{AC} brain per ventricle ratio at the anterior commissure, *BVR_{PC}* brain per ventricle ratio at the posterior commissure, *CI* confidence interval, *EI* Evans index, *iNPH* idiopathic normal pressure hydrocephalus, *N* number of persons within each MRI category examined for the corresponding clinical feature of NPH, *n* number of persons within each MRI category with the corresponding clinical feature of NPH, *z-EI* z-Evans index

*$P < 0.05$, **$P < 0.01$, ***$P < 0.001$

Table A2. Comparison of volumetric measurements between iNPH$_{Possible}$ and non-iNPH groups

	iNPH$_{Possible}$ (n=9), median *vRaw; vNorm*	IQR *(vRaw); (vNorm)*	95% CI *(vRaw); (vNorm)*	Non-iNPH (n=776), median *vRaw; vNorm*	IQR *(vRaw); (vNorm)*	95% CI *(vRaw); (vNorm)*	Δ Volume difference, iNPH$_{Possible}$ vs non-iNPH, median
**Left+right lateral ventricles (mL)**	69.3***; 60.1***	(55.8-84.4); (59.1-70.6)	(55.8-79.9); (59.1-67.8)	30.4; 31.8	(22.4-64.0); (25.3-57.9)	(29.0-31.3); (31.2-33.2)	38.9; 28.3
**Left+right lateral inferior ventricles (mL)**	2.9***; 2.8***	(2.2-3.9); (2.2-3.5)	(2.2-3.2); (2.2-3.2)	1.1; 1.2	(0.8-2.9); (0.9-2.5)	(1.1-1.2): (1.7-1.8)	1.8; 1.6
**3rd ventricle (mL)**	2.5***; 2.3**	(2.1-3.7); (2.1-3.5)	(2.1-3.4); (2.1-3.3)	1.7; 1.8	(1.3-3.0); (1.4-2.8)	(1.6-1.8); (1.8-1.9)	0.8; 0.5
**4th ventricle (mL)**	2.2**; 2.2*	(2.2-6.8); (2.0-6.6)	(2.2-2.9); (2.0-2.7)	1.8; 1.8	(1.5-2.9); (1.5-2.8)	(1.8-1.9); (1.8-1.9)	0.4; 0.4
**Brainstem (mL)**	21.4; 19.1	(19.7-23.8); (18.1-22.5)	(19.7-22.4); (18.1-22.2)	20.9; 21.1	(19.4-25.1); (29.6-65.3)	(20.1-21.1); (35.9-37.7)	2.3; 2
**Total ventricular**	76.6***; 69.2***	(66.6-91.7);	(66.6-86.9);	35.0; 36.9	(26.6-72.2);	(33.8-36.0);	42.6; 32.3

**CSF (mL)**		64.2-78.1)	(64.2-73.9)		(29.6-65.3)	(35.9-37.7)	
**Estimated total intracranial volume (mL)**	1679.1; NA	(1432.8-1933.7)	(1432.8-1711.6)	1488.2; NA	(1386.1-1781.1)	(1474.5-1499.3)	190.9
**Posterior corpus callosum (mL)**	1.05; 1.05	(0.95-1.4); 0.95-1.4);	(0.95-1.2); (0.95-1.2)	1.0; 1.0	(0.9-1.3); (0.9-1.3)	(0.98-1.02); (0.98-1.02)	0

CI confidence interval, *CSF* cerebrospinal fluid, *iNPH* idiopathic normal pressure hydrocephalus, $iNPH_{Possible}$ Possible iNPH according to modified International Guidelines*, IQR* Interquartile range 25th–75th percentiles, *vRaw* raw volumes, *vNorm* volumes normalized to intracranial volume

*$P < 0.05$, **$P < 0.01$, ***$P < 0.001$

Table A3. Correlations between morphological imaging markers and symptoms in persons with $iNPH_{Radiol}$

	Gait 6m (m)	Gait 30m self selected pace	Rombergs Open Eyes	Rombergs Closed Eyes	Gait Disturbance	Gait and/or balance disturbance	MMSE	Immidiate recall	Delayed recall	Digit span forward	Digit span backwar	Current dizziness	Dizziness/balance problems the last three months	Number of falling accidents	One leg static balance test	Cognitive impairment	Urinary Incontinence
**EI**	0,157	0,025	0,086	0,044	0,298	0,051	0,054	-0,126	-0,039	-0,075	0,074	0,057	0,013	0,050	0,070	0,095	-0,103
**Third Ventricle width (mm)**	-0,120	0,016	-0,114	-0,164	.329*	0,170	-0,229	-0,275	-.313*	-0,201	0,024	-0,057	-0,032	0,069	-0,092	0,203	-0,160
**Temporal horns mean (mm)**	-0,164	0,106	-0,169	-0,235	0,279	0,238	-.334*	**-.486****	**-.434****	-0,299	-0,252	0,084	-0,133	-0,012	-0,232	0,149	-0,035
**Fourth Ventricle (mm)**	0,018	-0,226	-0,040	0,216	-0,023	-0,090	0,021	-0,225	-0,116	-0,112	-0,204	0,031	0,095	0,028	-0,074	-0,028	-0,061
**Pons (mm)**	0,040	0,022	0,070	-0,134	-0,116	-0,029	0,089	0,179	0,250	-0,210	0,059	-0,123	0,098	**-.409****	-0,201	0,035	-0,070
**Midbrain (mm)**	0,042	-0,293	0,103	0,164	-0,205	-0,083	0,016	0,070	0,065	0,147	0,117	-0,282	-.332*	-0,062	0,126	0,118	-0,035
**z-EI**	-0,016	0,193	-0,169	-0,214	-0,004	-0,007	-0,001	0,052	0,081	0,060	0,183	0,146	-0,114	-0,128	-0,158	0,097	-0,162
**BVR_{AC}**	-0,103	-0,114	0,056	0,093	0,062	0,104	-0,037	-0,002	-0,056	-0,040	-0,183	-0,120	0,082	0,157	0,134	-0,066	0,230
**BVR_{PC}**	-0,053	-0,065	0,029	0,157	0,058	0,138	-0,043	0,013	0,059	-0,098	-0,155	-0,052	0,189	0,184	0,137	-0,043	0,142
**Callosal Angle (degrees)**	0,052	-0,101	0,065	0,113	-0,164	-0,070	0,069	0,053	0,134	0,122	-0,154	0,037	0,272	-0,005	-0,068	-0,052	0,184
**Compression of sulci n/N**	-0,041	0,065	.334*	0,193	-0,026	-0,168	-0,008	0,003	-0,082	-0,113	-0,035	-0,007	-0,041	-0,041	0,095	0,082	-0,303
**Enlarged sylvian fissure n/N**	-0,109	0,247	-0,285	-0,173	0,285	0,135	-0,150	-0,245	-0,243	-0,003	0,020	0,083	-0,151	0,203	-0,124	0,206	0,005

Abbreviations: AUC area under the curve, *BVR_{AC}* brain per ventricle ratio at the anterior commissure, *BVR_{pc}* brain per ventricle ratio at the posterior commissure, *CI* confidence interval, *EI* Evans index, *iNPH* idiopathic normal pressure hydrocephalus, *$iNPH_{Radiol}$* Radiologically probable iNPH according to modified International Guidelines, *N* number of persons within each MRI category examined for the corresponding clinical feature of NPH, *n* number of persons within each MRI category with the corresponding clinical feature of NPH, *z-EI* z-Evans index

Table A4. Correlations between morphological imaging markers and symptoms in the whole sample

	**Gait 6m (m)**	**Gait 30m self selected pace**	**Rombergs Open Eyes**	**Rombergs Closed Eyes**	**Gait Disturbance**	**Gait and/or balance disturbance**	**MMSE**	**Immidiate recall**	**Delayed recall**	**Digit span forward**	**Digit span backwar**	**Current dizziness**	**Dizziness/balance problems the last three months**	**Number of falling accidents**	**One leg static balance test**	**Cognitive impairment**	**Urinary incontinence**
**EI**	-.075*	0,071	-0,066	-.161**	.118**	.113**	-.074*	-.174**	-.172**	-0,017	-0,034	-0,021	-0,021	.082*	-.095*	.073*	0,006
**Third Ventricle width (mm)**	-.122**	.135**	-.099**	-.173**	.164**	.155**	-.074*	-.222**	**-.242****	-0,054	-0,059	-0,010	-0,023	.091*	-.118**	.113**	0,011
**Temporal horns mean (mm)**	-0,041	.109**	-0,051	-.084*	.081*	.080*	-.091*	-.142**	-.149**	-0,055	-.079*	-0,007	-0,022	.085*	-.079*	.109**	0,000
**Fourth Ventricle (mm)**	0,015	-0,014	-0,033	-0,054	.073*	0,065	-0,002	-0,032	-0,037	-0,004	0,002	-0,010	-0,007	0,068	0,003	0,033	0,003
**Pons (mm)**	.123**	-.073*	.073*	0,015	-.083*	-.079*	.090*	0,040	0,029	0,063	.080*	0,015	0,016	-0,005	.082*	-0,017	-0,043
**Midbrain (mm)**	.133**	-.127**	.100**	.072*	-0,057	-.089*	.079*	0,064	.086*	.110**	0,060	-0,003	0,006	-0,034	.133**	-0,043	-0,012
**z-EI**	-0,045	.081*	-.089*	-.128**	.113**	.113**	-0,030	-.090*	-.093**	-0,002	-0,017	0,015	-0,030	.099**	-.096**	0,068	0,025
**BVR_{AC}**	0,052	-.077*	.090*	.130**	-.114**	-.106**	0,020	.103**	.106**	0,003	0,028	-0,030	0,013	-.082*	.088*	-0,054	-0,003
**BVR_{PC}**	.102**	-.091*	.086*	.114**	-.139**	-.129**	0,026	.123**	.114**	0,034	0,041	0,018	0,041	-.100**	.091*	-0,054	-0,032
**Callosal Angle (degrees)**	0,028	-0,046	0,030	0,003	-.093**	-0,046	0,011	.094**	.100**	0,029	0,018	0,010	0,050	-0,051	0,050	-.086*	0,000
**Compression of sulci n/N**	-0,057	0,044	0,012	-0,047	0,003	0,010	-0,006	-0,022	-0,040	-0,018	-0,028	-0,002	-0,029	-0,008	-0,033	0,066	-0,021

**Enlarged sylvian fissure n/N**	-.095**	.081*	-0,063	-0,054	.103**	.087*	-0,035	-0,048	-.076*	-0,008	-0,053	0,007	-0,045	0,044	-0,054	.083*	0,031

Abbreviations: AUC area under the curve, BVR_{AC} brain per ventricle ratio at the anterior commissure, BVR_{pC} brain per ventricle ratio at the posterior commissure, *CI* confidence interval, *EI* Evans index, *iNPH* idiopathic normal pressure hydrocephalus, *N* number of persons within each MRI category examined for the corresponding clinical feature of NPH, *n* number of persons within each MRI category with the corresponding clinical feature of NPH, *z-EI* z-Evans index
**P* < 0.05, ***P* < 0.01, ****P* < 0.001

Table A5. Correlations between raw volumetric measurements and symptoms in persons with $iNPH_{Radiol}$

	**Gait 6m (m)**	**Gait 30m self selected pace**	**Rombergs Open Eyes**	**Rombergs Closed Eyes**	**Gait Disturbance**	**Gait and/or balance disturbance**	**MMSE**	**Immidiate recall**	**Delayed recall**	**Digit span forward**	**Digit span backwar**	**Current dizziness**	**Dizziness/balance problems the last three months**	**Number of falling accidents**	**One leg static balance test**	**Cognitive impairment**	**Urinary incontinence**
**Left + right lateral ventricles (mL)**	0,108	-0,060	0,096	-0,109	0,023	-0,269	-0,084	-0,011	-0,037	-0,199	0,035	-0,078	-0,049	-0,196	0,030	0,121	-0,254
**Left + right lateral inferior ventricles (mL)**	-0,079	0,170	-0,243	-0,142	0,195	0,088	-0,148	-0,205	-0,163	-0,068	0,230	0,201	0,032	-0,137	-0,205	0,020	-0,237
**Third Ventricle (mL)**	0,064	-0,149	0,113	0,037	0,113	-0,128	-0,089	-0,105	-0,089	-0,111	-0,044	**-.382***	-0,146	-0,116	0,102	0,155	-0,225
**Fourth Ventricle (mL)**	0,043	-0,107	-0,249	0,201	-0,137	-0,040	-0,070	-0,265	-0,015	-0,140	-0,102	-0,142	0,024	0,050	-0,028	0,061	-0,110
**Brainstem (mL)**	0,243	-0,277	0,074	0,049	-0,138	-0,236	0,090	0,083	0,120	-0,233	0,036	-0,181	0,016	**-.414***	-0,084	0,013	-0,110
**Total ventricular CSF (mL)**	0,108	-0,063	0,096	-0,101	0,004	-0,263	-0,111	-0,044	-0,057	-0,216	0,038	-0,097	-0,073	-0,237	0,021	0,128	-0,271
**Estimated total intracranial volume (mL)**	0,060	-0,095	-0,007	-0,098	0,004	-0,148	-0,172	-0,035	-0,017	-.373*	-0,044	-0,058	-0,081	-0,299	0,014	0,074	**-.416***
**Corpus Callosum posterior (mL)**	0,033	-0,137	0,032	-0,069	-0,082	-0,108	0,015	-0,043	-0,021	0,149	-0,130	0,045	-0,146	-0,002	0,076	0,148	0,006

CSF cerebrospinal fluid, *iNPH* idiopathic normal pressure hydrocephalus, $iNPH_{Radiol}$ Radiologically probable iNPH according to modified International Guidelines *MMSE* Mini mental state exam, *vRaw* raw volumes, *vNorm* volumes normalized to intracranial volume
* = *p* < 0.05; ** = *p* < 0.01; *** p<0.001

Table A6. Correlations between raw volumetric measurements and symptoms in the whole sample

	Gait 6m (m)	Gait 30m self selected pace	Rombergs Open Eyes	Rombergs Closed Eyes	Gait Disturbance	Gait and/or balance disturbance	MMSE	Immidiate recall	Delayed recall	Digit span forward	Digit span backwar	Current dizziness	Dizziness/balance problems the last three months	Number of falling accidents	One leg static balance test	Cognitive impairment	Urinary incontinence
**Left + right lateral ventricles (mL)**	-0,069	0,049	-.086*	-.157**	.099**	.080*	-0,022	-.134**	-.139**	0,027	-0,015	-0,046	-0,052	.082*	-.097**	0,052	-0,022
**Left + right lateral inferior ventricles (mL)**	-.075*	.084*	-0,062	-.089*	.092*	.075*	-0,038	-.123**	-.143**	-0,035	-0,036	-0,035	-0,049	.074*	-.121**	.091*	-0,036
**Third Ventricle (mL)**	-0,059	.074*	-0,054	-.131**	.088*	.083*	-0,021	-.181**	-.200**	-0,046	-0,048	-0,030	-0,040	0,064	-.080*	.076*	-0,025
**Fourth Ventricle (mL)**	.084*	-0,032	-0,041	-0,053	0,059	0,058	0,005	-.085*	-.100**	-0,002	0,008	-0,007	-0,009	0,045	-0,014	0,027	-0,014
**Brainstem (mL)**	**.283****	-.155**	.101**	0,053	-0,057	-.091*	.087*	-0,055	-.074*	.099**	.124**	-0,056	-0,068	-0,036	.163**	-0,035	-.114**
**Total ventricular CSF (mL)**	-0,066	0,051	-.085*	-.156**	.103**	.087*	-0,023	-.140**	-.147**	0,021	-0,016	-0,043	-0,051	.083*	-.100**	0,054	-0,023
**Estimated total intracranial volume (mL)**	.177**	-.094**	-0,003	-0,040	0,034	-0,007	0,038	-.132**	-.161**	.101**	.097**	-.097**	-.089*	-0,007	0,049	-0,016	-.117**
**Corpus Callosum posterior (mL)**	0,056	-.095**	0,011	-0,038	-0,024	-.075*	0,064	0,006	0,005	.106**	.079*	-0,016	-0,049	-0,004	0,045	-0,057	-0,057

CSF cerebrospinal fluid, *iNPH* idiopathic normal pressure hydrocephalus, *MMSE* Mini mental state exam, *vRaw* raw volumes, *vNorm* volumes normalized to intracranial volume
* = $p < 0.05$; ** = $p < 0.01$; *** $p<0.001$

Table A7. Correlations between normalized volumetric measurements and symptoms in persons with $iNPH_{Radiol}$

	Gait 6m (m)	Gait 30m self selected pace	Rombergs Open Eyes	Rombergs Closed Eyes	Gait Disturbance	Gait and/or balance disturbance	MMSE	Immidiate recall	Delayed recall	Digit span forward	Digit span backwar	Current dizziness	Dizziness/balance problems the last three months	Number of falling accidents	One leg static balance test	Cognitive impairment	Urinary incontinence
**Left + right lateral ventricles (mL)**	0,018	0,027	0,036	-0,164	0,082	-0,128	0,027	0,011	-0,045	0,070	0,061	0,019	-0,049	-0,063	-0,065	0,034	-0,012
**Left + right lateral inferior ventricles (mL)**	-0,157	0,234	-0,279	-0,125	0,195	0,168	-0,099	-0,218	-0,193	0,003	0,186	0,272	0,065	-0,057	-0,327	-0,027	-0,087
**Third Ventricle (mL)**	-0,052	-0,092	0,034	0,026	0,193	0,034	0,035	-0,055	-0,014	0,113	-0,033	**-.356***	-0,129	0,070	0,045	0,094	0,000
**Fourth Ventricle (mL)**	-0,031	-0,111	-0,215	0,226	-0,171	-0,013	0,002	-0,226	0,027	-0,021	-0,110	-0,104	0,065	0,091	-0,042	0,013	0,040
**Brainstem (mL)**	0,248	-0,275	0,000	0,098	-0,205	-0,175	0,290	0,153	0,169	0,037	0,055	-0,110	0,146	-0,191	-0,169	-0,094	0,323
**Total ventricular CSF (mL)**	-0,013	0,067	-0,013	-0,193	0,092	-0,094	0,017	0,031	-0,030	0,044	0,086	0,006	-0,024	-0,074	-0,100	0,040	-0,012
**Estimated total intracranial volume (mL)**	0,060	-0,095	-0,007	-0,098	0,004	-0,148	-0,172	-0,035	-0,017	**-.373***	-0,044	-0,058	-0,081	-0,299	0,014	0,074	**-.416***
**Corpus Callosum posterior (mL)**	0,033	-0,137	0,032	-0,069	-0,082	-0,108	0,015	-0,043	-0,021	0,149	-0,130	0,045	-0,146	-0,002	0,076	0,148	0,006

CSF cerebrospinal fluid, *iNPH* idiopathic normal pressure hydrocephalus, $iNPH_{Radiol}$ Radiologically probable iNPH according to modified International Guidelines, *MMSE* Mini mental state exam, *vRaw* raw volumes, *vNorm* volumes normalized to intracranial volume

* = $p < 0.05$; ** = $p < 0.01$; *** $p<0.001$

Table A8. Correlations between normalized volumetric measurements and symptoms in the whole sample

	Gait 6m (m)	Gait 30m self selected pace	Rombergs Open Eyes	Rombergs Closed Eyes	Gait Disturbance	Gait and/or balance disturbance	MMSE	Immidiate recall	Delayed recall	Digit span forward	Digit span backwar	Current dizziness	Dizziness/balance problems the last three months	Number of falling accidents	One leg static balance test	Cognitive impairment	Urinary incontinence
**Left + right lateral ventricles (mL)**	**-.235****	.157**	-.136**	-.168**	.094**	.107**	-0,062	-0,059	-0,056	-0,061	-.097**	0,027	0,018	.106**	-.178**	.091*	.082*
**Left + right lateral inferior ventricles (mL)**	-.197**	.153**	-.107**	-.093*	.080*	.089*	-.088*	-0,056	-.071*	-.102**	-.100**	0,034	0,018	.084*	-.184**	.139**	0,021
**Third Ventricle (mL)**	-.183**	.148**	-.085*	-.144**	.086*	.112**	-0,059	-.134**	-.144**	-.117**	-.123**	0,027	0,008	.086*	-.124**	.115**	0,037
**Fourth Ventricle (mL)**	0,044	-0,006	-0,052	-0,035	0,046	0,059	-0,006	-0,040	-0,048	-0,033	-0,019	0,030	0,025	0,045	-0,018	0,034	0,021
**Brainstem (mL)**	**.235****	-.142**	.133**	.107**	-.089*	-.114**	0,066	0,053	0,044	0,052	.089*	0,015	-0,016	-0,038	.187**	-0,023	-0,055
**Total ventricular CSF (mL)**	-.234**	.157**	-.137**	-.168**	.100**	.112**	-0,065	-0,065	-0,065	-0,069	-.102**	0,030	0,021	.108**	-.181**	.096**	.080*
**Estimated total intracranial volume (mL)**	0,056	-.095**	0,011	-0,038	-0,024	-.075*	0,064	0,006	0,005	.106**	.079*	-0,016	-0,049	-0,004	0,045	-0,057	-0,057
**Corpus Callosum posterior (mL)**	-.235**	.157**	-.136**	-.168**	.094**	.107**	-0,062	-0,059	-0,056	-0,061	-.097**	0,027	0,018	.106**	-.178**	.091*	.082*

CSF cerebrospinal fluid, *iNPH* idiopathic normal pressure hydrocephalus, *MMSE* Mini mental state exam, *vRaw* raw volumes, *vNorm* volumes normalized to intracranial volume
* = $p < 0.05$; ** = $p < 0.01$; *** p<0.001

Table A9. Correlations between normalized and raw volumetric measurements, imaging markers and the iNPH index in persons with $iNPH_{Radiol}$ and within the whole sample

	**iNPH index**<br>**Spearman correlation coefficent**<br>*$iNPH_{Radiol}$*	**iNPH index**<br>**Spearman correlation coefficent**<br>*Whole sample*
**Left + right lateral ventricles (mL)** *vNorm*	0,053	-.168**
**Left + right lateral inferior ventricles (mL)** *vNorm*	-0,202	-.156**
**Third Ventricle (mL)** *vNorm*	0,104	-.126**
**Fourth Ventricle (mL)** *vNorm*	0,175	-0,019
**Brainstem (mL)** *vNorm*	-0,149	.157**
**Total ventricular CSF (mL)** *vNorm*	0,034	-.169**
**Corpus Callosum posterior (mL)** *vNorm*	0,216	.096**
**Left + right lateral ventricles (mL)** *vRaw*	0,242	-0,026
**Left + right lateral inferior ventricles (mL)** *vRaw*	-0,066	-0,054
**Third Ventricle (mL)** *vRaw*	0,248	-0,034
**Fourth Ventricle (mL)** *vRaw*	0,244	0,020
**Brainstem (mL)** *vRaw*	0,077	.206**
**Total ventricular CSF (mL)** *vRaw*	0,242	-0,028
**Estimated total intracranial volume (mL)** *vRaw*	0,205	.147**
**Corpus Callosum posterior (mL)** *vRaw*	0,216	.096**
**EI**	0,018	-.070*
**Third Ventricle width (mm)**	-0,038	-.077*
**Temporal horns mean (mm)**	-0,112	-.081*
**Fourth Ventricle (mm)**	0,181	0,002
**Pons (mm)**	-0,107	.090*
**Midbrain (mm)**	0,159	.114**
**z-EI**	0,053	-0,068
**BVR_{AC}**	-0,085	0,048
**BVR_{PC}**	-0,032	.072*
**Callosal Angle (degrees)**	-0,023	0,039
**Compression of sulci (yes/no)**	0,272	-0,014
**Enlarged sylvian fissure (yes/no)**	-0,132	-.070*

BVR_{AC} brain per ventricle ratio at the anterior commissure, *BVR_{pC}* brain per ventricle ratio at the posterior commissure, *CI* confidence interval, *CSF* cerebrospinal fluid, *EI* Evans index, *iNPH* idiopathic normal pressure hydrocephalus, *$iNPH_{Radiol}$* Radiologically probable iNPH according to modified International Guidelines, *MMSE* Mini mental state exam, *vRaw* volumes, *vNorm* volumes normalized to intracranial volume, *z-EI* z-Evans index

*$P < 0.05$, **$P < 0.01$, ***$P < 0.001$

Table A10. Comparison of imaging markers and volumetric measurements between men and women in the $iNPH_{Radiol}$ group

	**Men (n=28)** mean/median	**Women (n=12)** mean/median
**Evans Index (mm)** mean/median	0.33/0.32	0.33/0.33
**Z-EI** mean/median	0.35/0.35	0.35/0.35
**Third ventricle (mm)** mean/median	10.46/10.45*	8.16/7.95
**Temporal horn width (mm)** mean/median	6.17/6.55	4.78/3.7
**BVR_{AC}** mean/median	1.19/1.13	1.24/1.18
**BVR_{PC}** mean/median	1.67/1.59	1.75/1.60
**Callosal angle (degrees)** mean/median	93.31/92.40	101.15/104.80
**The maximum diameter of the midbrain (mm)** mean/median	11.65/11.60	11.63/11.50
**The maximum diameter of the pons (mm)** mean/median	23.37/23.30	22.96/23.10
**The maximum diameter of the fourth ventricle (mm)** mean/median	11.43/11.65	11.60/11.30
**Compression of sulci** n/N	6/28	1/12
**Enlarged sylvian fissure right and/or left** n/N	10/28	4/12

BVR_{AC} brain per ventricle ratio at the anterior commissure, *BVR_{PC}* brain per ventricle ratio at the posterior commissure, *CI* confidence interval, *EI* Evans index, *iNPH* idiopathic normal pressure hydrocephalus, *N* number of persons within each MRI category examined for the corresponding clinical feature of NPH, *n* number of persons within each MRI category with the corresponding clinical feature of NPH, *z-EI* z-Evans index

*$P < 0.05$, **$P < 0.01$, ***$P < 0.001$

Table A11. Comparison of imaging markers and volumetric measurements between men and women in the $iNPH_{Radiol}$ group

	Men (n=28), median *vRaw; vNorm*	IQR *(vRaw); (vNorm)*	95% CI *(vRaw); (vNorm)*	Women (n=12, median *vRaw; vNorm*	IQR *(vRaw); (vNorm)*	95% CI *(vRaw); (vNorm)*	Δ Volume difference, men vs women, median
**Left+right lateral ventricles (mL)**	72.2; 60.1	(59.8-85.3); (48.0-68.4)	(65.8-81.1); (55.2-67.6)	55.8; 61.3	(41.8-73.5); (46.5-71.8)	(41.8-73.5); (46.5-71.8)	16.4; 1.2
**Left+right lateral inferior ventricles (mL)**	3.2; 2.8	(2.7-4.4); (2.1-4.1)	(2.9-4.1): (2.2-3.5)	2.1; 2.3	(1.3-3.2); (1.5-2.9)	(1.3-3.2); (1.5-2.9)	1.1; 0.5
**3rd ventricle (mL)**	3.2*; 2.6	(2.4-3.6); (1.9-3.2)	(2.5-3.4); (2.0-3.0)	2.3; 2.4	(1.9-2.7); (2.1-2.8)	(1.9-2.7); (2.1-2.8)	0.9; 0.2
**4th ventricle (mL)**	2.6; 2.2	(2.1-2.7); (1.7-2.6)	(2.2-2.5); (1.8-2.5)	2.2; 2.1	(1.8-2.8); (1.8-2.9)	(1.8-2.8); (1.8-2.9)	0.4; 0.1
**Brainstem (mL)**	21.4*; 19.1	(19.3-22.5); (17.6-19.9)	(20.6-22.3); (17.9-19.7)	19.2; 20.2	(17.1-21.5); (17.5-21.7)	(17.1-21.5); (17.5-21.7)	2.2; 1.1
**Total ventricular**	82.5-67.2	(68.0-94.0);	(75.0-89.3);	66.6; 69.3	(45.9-82.6);	(45.9-82.6);	15.9; 2.1

**CSF (mL)**		(57.0-78.6)	(63.2-73.9)		51.0-80.3)	(51.0-80.3)	
**Estimated total intracranial volume (mL)**	1707.1***; NA	(1648.2-1812.4)	(1669.3-1750.5)	1455.8; NA	(1403.7-1571.7)	(1403.7-1571.7)	251,3
**Posterior corpus callosum (mL)**	1.05;1.05	(0.75-1.14); (0.75-1.14)	(0.92-1.1); (0.92-1.1)	1.09; 1.09	(1.1-1.2); 1.1-1.2);	(1.1-1.2); (1.1-1.2)	0.04

CI confidence interval, *CSF* cerebrospinal fluid, *iNPH* idiopathic normal pressure hydrocephalus, , *iNPH$_{Radiol}$* Radiologically probable iNPH according to modified International Guidelines*, IQR* Interquartile range 25th–75th percentiles, *vRaw* raw volumes, *vNorm* volumes normalized to intracranial volume
**P* < 0.05, ***P* < 0.01, ****P* < 0.001

Table A12. Comparison International and Japanese Guidelines for iNPH diagnosis

	**Possible iNPH**	**Probable iNPH**	**Definite iNPH**
**International**	1. May have a subacute or	1. Insidious onset of	

**Guidelines**	indeterminate onset; begin at any age; have lasted less than 3 m or indeterminately; follow conditions that are unlikely causally related; non- or not clearly progressive; not entirely attributable to other conditions<br>2. ≥ 1 of gait disturbance, cognitive decline, urinary incontinence<br>3. EI > 0.30;<br>a. No obstruction to CSF flow;<br>b. Cerebral atrophy potentially explaining<br>c. ventricular size;<br>d. Structural lesions potentially influencing<br>e. ventriculomegaly are accepted<br>f. Opening pressure measurement not available or pressure outside the range of probable INPH	symptoms; after age of 40 y; duration of at least 3 to 6 months, no known cause; progressive over time; absence of other conditions that might explain symptoms<br>2. Gait disturbance **and** ≥ 1 of cognitive decline, urinary incontinence<br>3. EI >0.3 not entirely caused by atrophy; no obstruction of CSF flow and ≥ 1 of:<br>a. Enlargement of temporal horns not entirely caused by hippocampus atrophy<br>b. Callosal angle ≥40 degrees<br>c. Altered periventricular water content not attributable to arteriolosclerosis or demyelination<br>d. Flow void in aqueduct or 4th ventricle<br>e. CSF opening pressure 5-18mmg Hg (or 70–245 mm H2O)	
**Japanese Guidelines**	1. More than one symptom in the clinical triad (gait disturbance, cognitive decline, urinary incontinence)<br>2. Above mentioned clinical symptoms cannot completely be explained by other neurological or non-neurological disease.<br>3. Preceding diseases possibly causing ventricular dilation (including subarachnoidal hemorrhage, meningitis, head injury, congenital/developmental hydrocephalus, and aqueductal stenosis) are not obvious.<br>4. 60 years or older<br>5. Dilated Ventricles on CT/MRIs (Evans Index >0.3)<br>6. Even if even if EI is <0.3, but the other indices meet the conditions of expanded inferior horn of the lateral ventricle, such as CA <90°, z-EI >0.42, BVR at AC level <1.0, and/or BVR at PC level <1.5, it is acceptable to diagnose possible iNPH.	1. Meets the requirements for possible iNPH<br>2. CSF pressure of 200 mm H2O or less and normal CSF content<br>3. One of the following two investigational features:<br>a) Neuroimaging features of narrowing of the sulci and subarachnoidal space over the high convexity/midline surface (DESH) with gait disturbance: small stride, shuffle, instability during walking, and increase in instability on turning<br>b) Improvement of symptoms after CSF tap test and/ or drainage test	Diagnosis of definite iNPH is made when objective improvement of symptoms is shown after shunt surgery.

CSF Cerebrospinal Fluid, *EI* Evans Index, *CA* Callosal Angle

Figure A1. Illustration of morphological radiological marker measurements

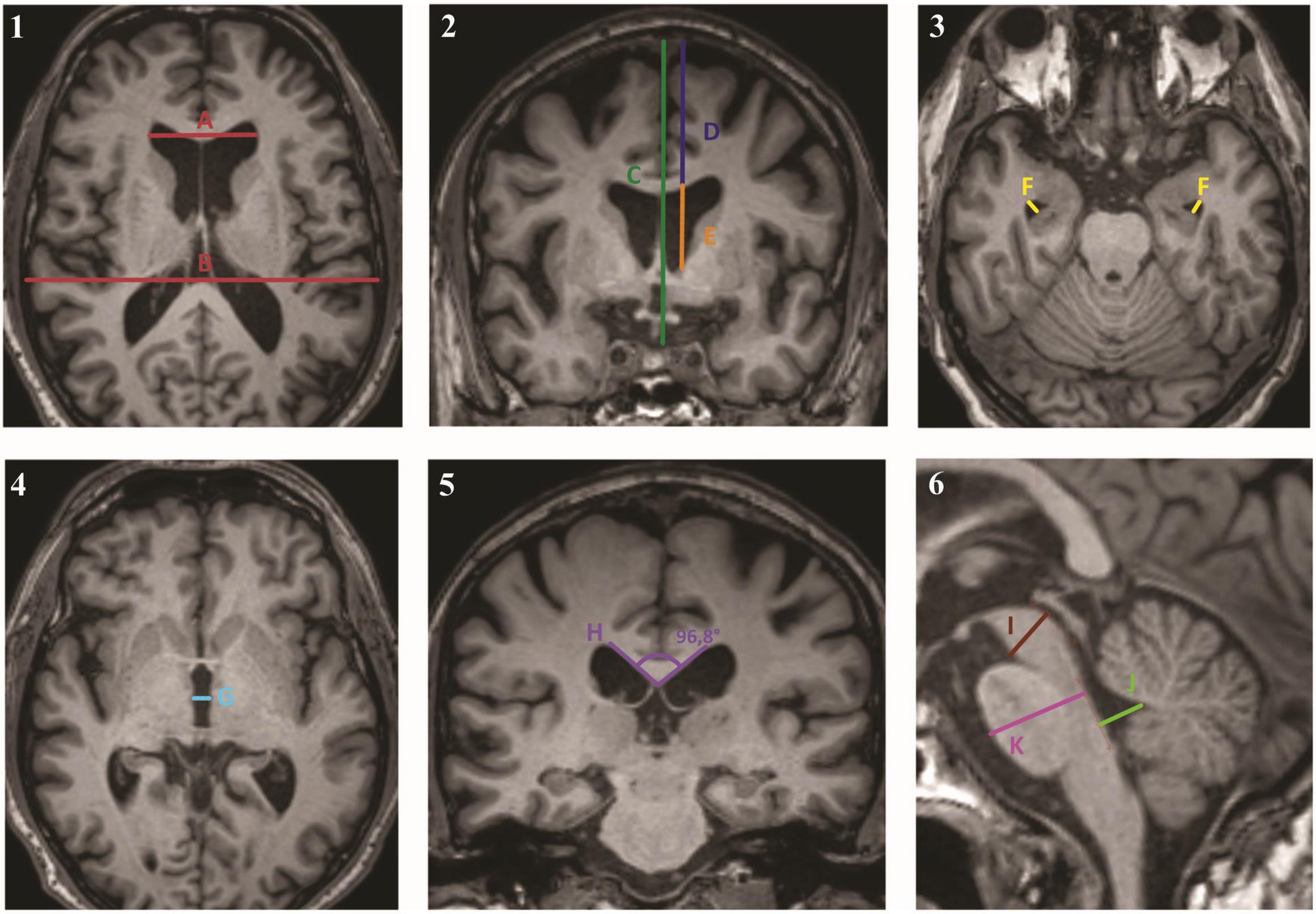

1. Evans Index (EI) was measured as the ratio between the maximum diameter of the frontal horns of the lateral ventricles (A) and the maximum inner diameter of the skull in the same transaxial slice (B). 2. The Z-Evans Index (z-EI) was measured as the ratio between the maximum z-distance of the frontal horns of the lateral ventricles (E) divided by the maximum cranial z-distance at the midline on the coronal plane on the AC (C). 2. The brain per ventricle ratios (BVRs) were defined as the maximum z-axial length of the brain just above the lateral ventricles (D), divided by the maximum length of the lateral ventricles (E) determined on the AC and PC level respectively (7). 3. The maximum diameter of the temporal horns, i.e. the temporal horn width (TWH) (F), was measured on transaxial images. Among 6 slices, centered around the mesencephalon-pons transitional zone, the slice with the largest TWH was chosen and the diameter of both horns was calculated in millimeters. The average of the bilateral measurements was registered. 4. The widest diameter of the third ventricle (G), was measured in millimeters in the center of the ventricle in the anteroposterior direction and in the widest part in the inferior-superior direction. 5.The callosal angle (CA) (H) was calculated between the lateral ventricles in the coronal plane intersecting the posterior commissure. 6. The maximum anterior-posterior diameter of the pons (K) was measured in millimeters in the mid-sagittal slice perpendicular to a reference line along the posterior aspect of the pons. The maximum anterior-posterior diameter of the fourth ventricle (J) was calculated in millimeters between the posterior range of the pons and the deepest part of the fourth ventricle in the mid-sagittal slice perpendicular to a reference line along the posterior aspect of the pons. The maximum anterior-posterior distance of the midbrain (I) was determined on a mid- sagittal image in millimeters perpendicular to a reference line along the cerebral aqueduct.
